# Evaluating the Tissue Optical Perfusion Pressure Method in Diabetic Patients with and Without Media Arterial Calcification

**DOI:** 10.3390/jcm15103891

**Published:** 2026-05-18

**Authors:** Igli Kalaja, Max Maria Meertens, Volker Hubert Schmitt, Birgit Linnemann, Gerhard Weißer, Melanie Schwaderlapp, Sarah Schneider, Leoni Hoffmann, Christine Espinola-Klein

**Affiliations:** 1 Department for Angiology—Center of Cardiology, University Medical Center of the Johannes Gutenberg University, 55131 Mainz, Germany; igli.kalaja@unimedizin-mainz.de (I.K.);; 2 Clinic III for Internal Medicine, Faculty of Medicine and University Hospital Cologne, University of Cologne, 50937 Cologne, Germany

**Keywords:** tissue optical perfusion pressure, ABI, TBI, oABI, puls wave index, media arterial calcification, diabetic mellitus

## Abstract

**Background**: The ankle-brachial index (ABI) is a popular method for evaluating peripheral artery disease (PAD). However, it is unreliable in patients with diabetes mellitus (DM), particularly in cases of media arterial calcification (MAC), where falsely elevated or unreliable values may be produced. The toe-brachial index (TBI) is therefore recommended in such cases, but has its limitations. The tissue optical perfusion pressure (TOPP) method is another automated diagnostic protocol combining oscillometric ABI measurement (oABI) and photo-plethysmographic pulse-wave assessment using the pulse wave index (PWI). The study evaluated TOPP-derived parameters in diabetic patients with or without MAC, in comparison with established functional vascular examinations. **Methods**: PAD patients with DM presenting in our outpatient clinic were enrolled prospectively from January to August 2024. Patients with peripheral bypasses or deemed unsuitable for the TOPP method were excluded. All patients received an ABI, TBI and TOPP measurement. **Results**: A total of 107 patients with DM were included in the present study. 38 patients presented with MAC and 69 patients without. The majority were male. Most patients presented with claudication (20 Fontaine stage IIa, 30 stage IIb), 9 presented with rest pain (Fontaine stage III), and 31 with wounds (Fontaine stage IV). 17 patients were free of symptoms (Fontaine stage I). The two parameters of the TOPP method, oABI and PWI, both correlated with the TBI and ABI. In patients with MAC, the oABI did not correlate with any other measurement, but the PWI did weakly correlate with the TBI. MAC is an important factor in influencing measurement accuracy. Despite their limitations, the TBI showed a significant correlation to the clinical symptoms (correlation coefficient = −0.387, *p* < 0.001). **Conclusions**: In patients without MAC, oABI and PWI correlated with ABI and TBI. TBI was the most reliable parameter in those with MAC. PWI correlated with TBI, but the correlation was weak. TBI should not be replaced by PWI. PWI may provide complementary information in a diagnostic protocol. oABI did not correlate with clinical symptom severity in DM patients, independently of the presence of MAC, and is unsuitable as a stand-alone parameter. A combination of TBI and TOPP-derived parameters may help to assess the severity of peripheral artery disease in diabetic patients with MAC. Larger multicentre studies are required.

## 1. Introduction

PAD is a highly prevalent condition in the elderly, with global estimates suggesting a current prevalence of approximately 200 million cases [1]. However, the significant number of underdiagnosed patients poses a substantial challenge in terms of the implementation of targeted and adequate treatment [2]. Consequently, the establishment of rapid and easy-to-perform diagnostic methods with high sensitivity is crucial in today’s healthcare settings.

ABI is a standardised test used in clinical practice to diagnose PAD. It is cost-efficient and has a sensitivity measured at rest of 68% to 84% with a specificity of 84% to 99%, making it the gold standard for diagnosing PAD today [1,3]. The ABI can be measured using Doppler as well as oscilometry. There is discordance in the current literature on whether the Doppler-guided method, which represents the gold standard, and the oABI are equivalent in the diagnosis of PAD [4]. Various parameters like age, gender, height, weight, body position and smoking were identified to influence these two measurement methods. In addition, problems with darker skin colour, high room temperatures, atrial fibrillation and patients with pacemakers can be observed during oABI measurement in detecting the pulse wave. Previous studies have reported that optical pulse-wave detection may be affected by technical and patient-related factors, including skin pigmentation, room temperature, arrhythmias and implanted pacemakers. Another key problem is the lack of standardisation in measurement technology. This includes patient positioning, cuff sizing, cuff positioning, the level of training among staff, and inconsistent pulse detection methods [1,5,6]. Both methods have limitations with regard to the circulatory diagnostics of the distal examination areas below the ankle joint, due to a lack of measuring location. This restricts the diagnosis of the peripheral perfusion of the foot tissue.

Another factor affecting the reliability of the ABI is MAC, which is commonly seen in patients with DM or kidney disease, a prevalent comorbidity in PAD patients. In patients with DM, the ABI is not reliable due to MAC, leading to a decreased sensitivity of the ABI measurement of only 35–73%, compared to 88–100% in patients without DM [7,8].

Since MAC is less prevalent in peripheral arteries such as the toe arteries, it is recommended to assess the peripheral perfusion in patients with DM by measuring the TBI [3,9,10], which has a sensitivity of 45% to 100% with a specificity of 17% to 100% [3].

The TOPP method is an automated evaluation of the peripheral perfusion, incorporating the oABI and the PWI [1]. It is important to note that the TOPP method does not constitute a single calculated index. Instead, it refers to an automated diagnostic protocol that combines oscillometric pressure measurement with photoplethysmographic pulse-wave assessment. In the present study, the two parameters derived from the TOPP protocol and utilised for analysis were the oABI and the PWI. The automation affects the reproducibility of the method, as there is no need to guarantee the same vascular expert will repeat the measurement when the patient returns for a follow-up examination.

Furthermore, patients with DM present a diagnostic challenge in terms of detecting the severity of PAD. Falsely elevated or unreliable ABI values are commonly seen in this population, which often suffers from MAC, leading to incompressible arteries. Therefore, the severity of PAD may be underestimated in patients with DM, even when established approaches such as the ABI are used [3,7,8].

In addition, TBI is recommended as an alternative diagnostic method for patients with suspected MAC. However, its diagnostic performance can vary considerably and may be limited by examiner dependency, toe wounds, previous amputations or severe diabetic foot complications [9,10]. Consequently, there is a clinical need for fast, standardised and reproducible diagnostic tools that can improve the assessment of peripheral perfusion in high-risk diabetic patients.

The TOPP method is expected to provide such an approach by combining oscillometric pressure measurements with photoplethysmographic pulse-wave assessment. However, the diagnostic value of this functional examination in diabetic patients suffering from PAD with and without MAC remains to be fully elucidated.

The present study, therefore, sought to evaluate the diagnostic value of the TOPP method in comparison with established functional vascular examinations, such as ABI and TBI, in patients with DM suffering from PAD and to assess the influence of MAC on the reliability of the measurement.

## 2. Materials and Methods

### 2.1. Ethical Approval

No additional approval for this study was needed, and this was granted by the ethics committee of the Medical Faculty of Johannes Gutenberg University, Mainz, Germany. Patient data was fully anonymised before the evaluation. No supplementary examinations, interventions, or measurements were performed for research purposes, and no identifiable personal data were collected, transferred, or disclosed.

### 2.2. Study Design

Between January 2024 and August 2024, diabetic patients who were admitted with proven or suspected PAD to our outpatient department were screened for examination using the TOPP Method. The diagnosis of PAD was made on the basis of clinical symptoms, previous vascular imaging, duplex ultrasound findings, and established guideline criteria in accordance with the 2024 European Society of Cardiology (ESC) guidelines [3].

The inclusion criteria comprised all patients aged over 18 years with DM, both with and without MAC. Patients who had already undergone previous vascular surgery (peripheral bypasses, TEVAR, EVAR and thrombendarterectomy) and those with a major amputation of the measured lower limb were excluded from the screening. In addition, all patients for whom no oABI measurement was possible (lipoedema, lymphoedema, wounds on cuff area, tremor and chronic pain syndrome) were excluded. To assess whether the TBI and the parameters of the TOPP method can distinguish between chronic limb-threatening ischemia (CLTI, stage Rutherford 4–6) and stable disease (stage Rutherford 1–3) and to assess the influence of MAC on the differentiation, the study population was first divided into these two clinical subgroups and subsequently stratified by the presence or absence of MAC.

The measurements were conducted by personnel with expertise in the field of functional diagnostics of peripheral blood flow. For the TOPP method measurements, an eight-channel oscillometry device was used (Angio Experience Pro 8; Sonotechnik, Maria Rain, Austria) with the simultaneous registration of two photoplethysmography (PPG) channels (Angio Experience Phlebo; Sonotechnik, Maria Rain, Austria) with the Software version (AngioExperiance 2.4.15). Furthermore, care was taken to ensure that the patients rested in a horizontal position for at least 5 min before each measurement in order to achieve a stable circulatory situation at rest.

### 2.3. Methods

The oscillometric system utilised for the examination (Angio Experience Pro 8; Sonotechnik, Maria Rain, Austria) had previously undergone validation [1,5].

#### 2.3.1. Ankle-Brachial Index Measurement

Following the application of the cuffs to the upper arms and ankles, the systolic pressure at the target vessels, the dorsalis pedis artery (DPA) and posterior tibial artery (PTA) was measured using a cw-Doppler probe after deflation of the ankle cuff, in accordance with the recommendation of the ESC and American Heart Association [3,6]. An ABI < 0.90 was considered pathological, while an ABI > 1.40 was interpreted as suggestive of MAC according to current guideline recommendations [3].

#### 2.3.2. Toe-Brachial Index-Measurement

Following the application of the cuffs to the upper arms and big toes, the PPG probes are attached to the big toes. The first derived pulse wave at the big toe is recorded acoustically and visually on the screen of the device by the examiner during automatic deflation of the cuffs, according to the recommendation of Høyer et al., 2013 and Singhania et al., 2024 [11,12]. A TBI < 0.70 was considered abnormal according to current guideline recommendations [3].

#### 2.3.3. Tissue Optical Perfusion Pressure-Measurement

**Figure 1:**
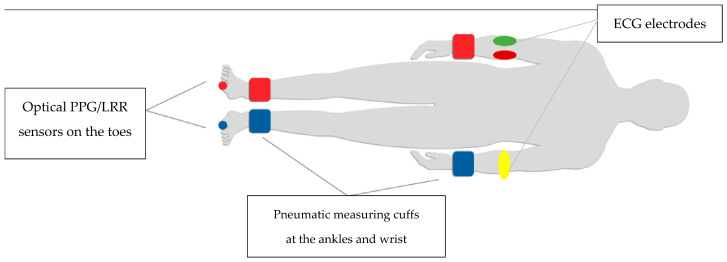
Schematic overview of the TOP measurement setup, including pneumatic cuffs, optical PPG/LRR sensors, and ECG electrodes.

For the purpose of this study, the TOPP method was defined as the automated measurement protocol that provided two separate parameters: oABI and PWI. The parameters were analysed separately, with TOPP not being regarded as a composite score. Following the application of the cuffs to the upper arms, wrists and ankles, and the electrocardiogram electrodes to the forearms, the PPGs are also applied to the big toes (Figure 1). The system exerts a pressure of 180 mmHg on all cuffs, which is reduced in steps of 20 mmHg, which was a modification on the cuff reduce speed according to the study of Horstick et al. 2020 and Youssef et al. 2020 [1,5].

#### 2.3.4. Media Arterial Calcification

MAC refers to calcium deposits in the medial layer of arterial walls without luminal narrowing [9]. In the functional arterial measurement, it is characterised by an ABI > 1.40 [3]. In the present study, MAC was defined functionally by an ABI > 1.40, as recommended by the current guidelines [3]. No additional imaging procedure, such as X-ray or ultrasound specifically aimed at confirming MAC, was performed systematically for study purposes. Whenever they were available, clinical imaging and duplex ultrasound findings were considered as part of the routine PAD assessment. However, they were not used as a standardised confirmatory criterion for the classification of MAC.

#### 2.3.5. Statistical Methods

Descriptive statistics of binary data are reported as absolute values, whereas continuous variables are expressed as mean ± standard deviation. The Shapiro–Wilk test was employed to ascertain whether the continuous variables exhibited a normal distribution. Depending on the distribution of the data, either parametric or non-parametric analyses were applied. The interpretation of correlation strength followed the recommendations set out by Cohen. The Spearman correlation was used to test for correlations. In case no legs were symptomatic or even both legs were symptomatic, both legs were included in the analysis. Comparing IC and CTLI patients with and without MAC, the median of the functional testing was compared using the Mann–Witney U Test. All statistical tests were performed using SPSS software (IBM SPSS Statistics 28, IBM Corp., Armonk, NY, USA).

No formal a priori power calculation was performed, as the study was designed as an exploratory prospective cohort study evaluating the diagnostic behaviour of TOPP-derived parameters in a real-world outpatient population.

## 3. Results

The present study included a total of 107 patients, all of whom had been diagnosed with diabetes mellitus. 38 patients presented with MAC, and 69 patients without MAC. Patients with MAC were significantly older (77.2 years vs. 69 years, *p* = 0.001). In both groups, most patients were male. The body surface area was comparable with 2.00 m^2^ in the group with MAC, and the group without MAC had a surface area of 1.97 m^2^. Most patients presented with claudication (20 Fontaine stage IIa, 30 stage IIb), 9 presented with rest pain (Fontaine stage III), and 31 with wounds (Fontaine stage IV). 17 patients were free from any symptoms (Fontaine stage I). The symptom severity differed between the groups (*p* = 0.033), as the largest share of patients in the MAC group was patients with Fontaine stage IV (42.1%), while in the group of patients without MAC, the largest share was patients with Fontaine Stage IIb (37.7%). Besides that, there were more patients in the MAC group who never smoked (52.6 vs. 30.5%, *p* = 0.012); no further differences were seen in the demographic data (Table 1). Also, no statistical differences were seen in the medication patients received, but patients with MAC took a Vitamin K antagonist more often (23.7% vs. 4.3%, *p* = 0.004, Table 2).

**Figure 2:**
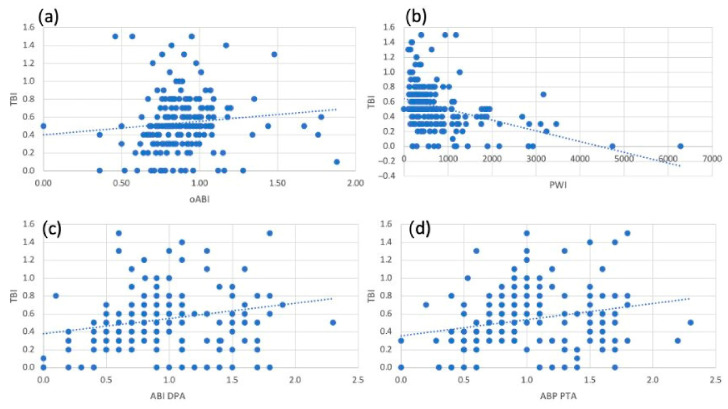
Scatter plot showing the correlation of the study population (**a**) between the Toe-Brachial-Index (TBI) and the oscillometric Ankle-Brachial-Index (oABI); (**b**) between the TBI and the Pulse-Wave-Velocity Index; (**c**) between the TBI and the Ankle-Brachial-Index (ABI) of the dorsalis pedis artery (DPA); (**d**) between the TBI and the ABI of the posterior tibialis artery (PTA).

**Figure 3:**
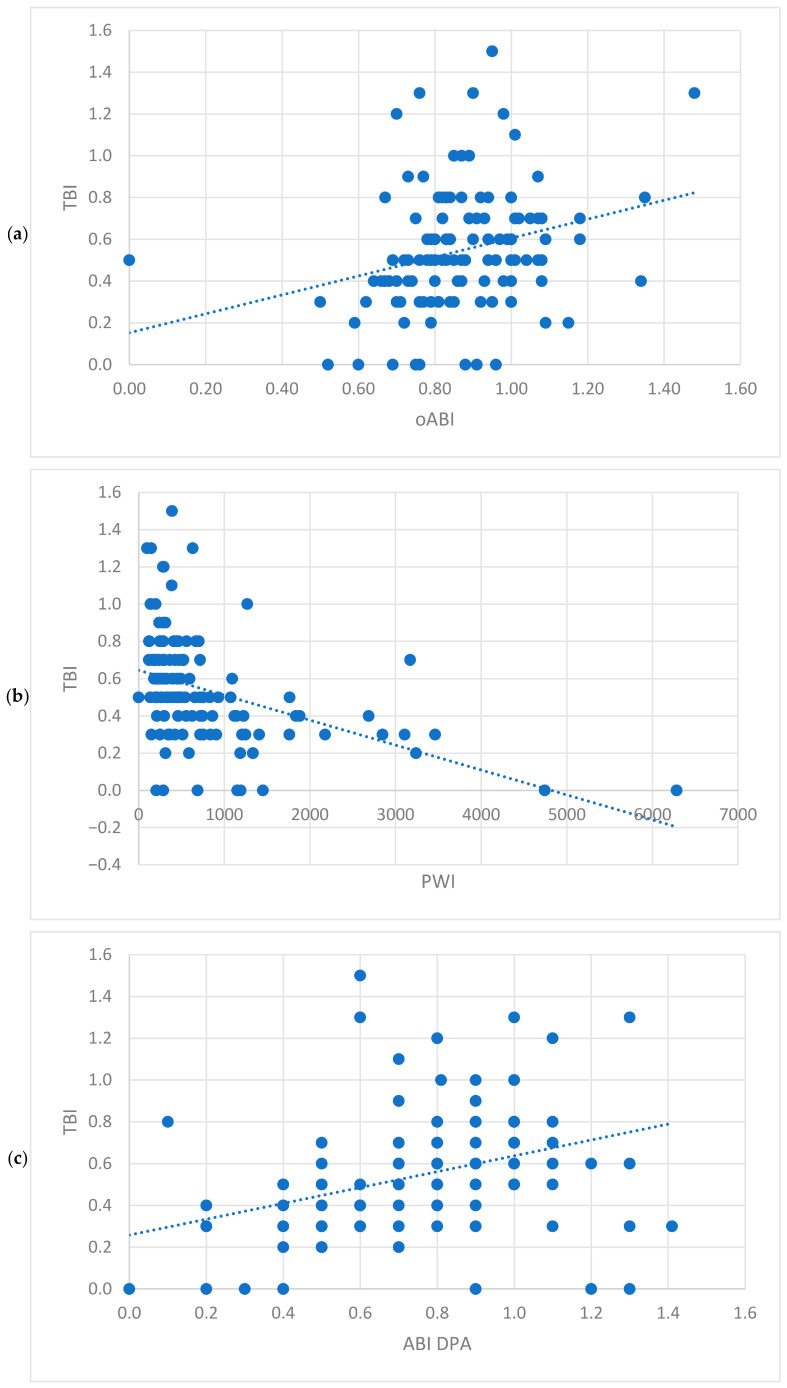

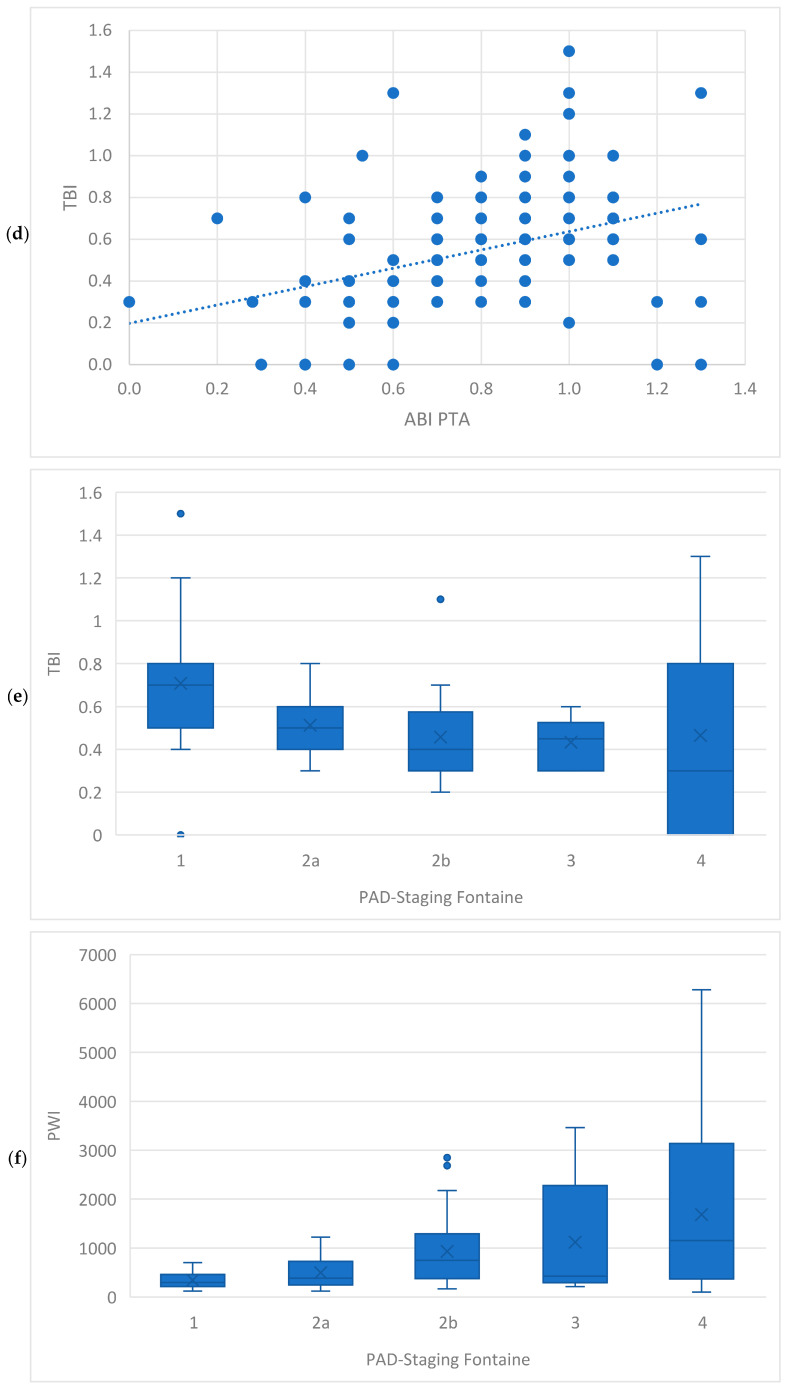

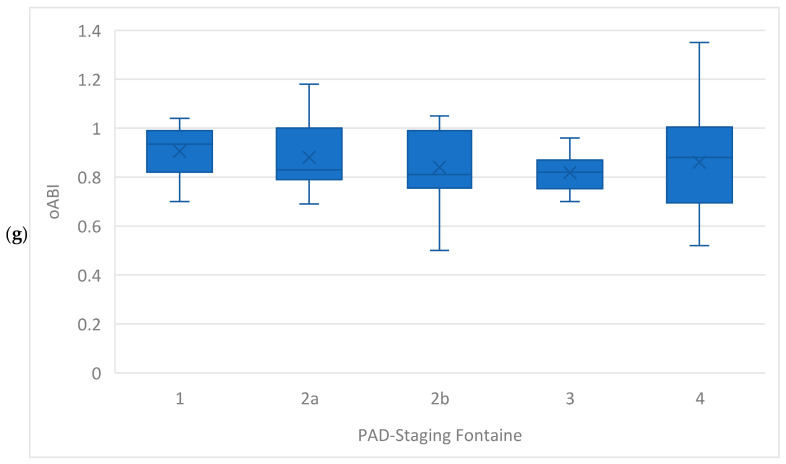
Scatter plot showing the correlation of the study population without media arterial calcification (**a**) between the Toe-Brachial-Index (TBI) and the oscillometric Ankle-Brachial-Index (oABI); (**b**) between the TBI and the Pulse-Wave-Velocity Index; (**c**) between the TBI and the Ankle-Brachial-Index (ABI) of the dorsalis pedis artery (DPA); (**d**) between the TBI and the ABI of the posterior tibialis artery (PTA) and Box-Plott-Diagram showing the correlation of the study population without media arterial calcification (**e**) between the TBI and the peripheral arterial disease staging according to Fontaine (PAD-Staging Fontaine); (**f**) between the PWI and the PAD-Staging Fontaine; (**g**) between the oABI and the PAD-Staging Fontaine.

**Figure 4:**
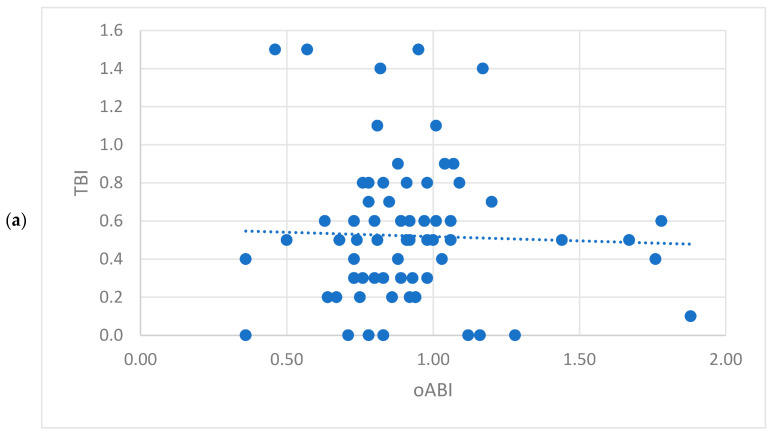

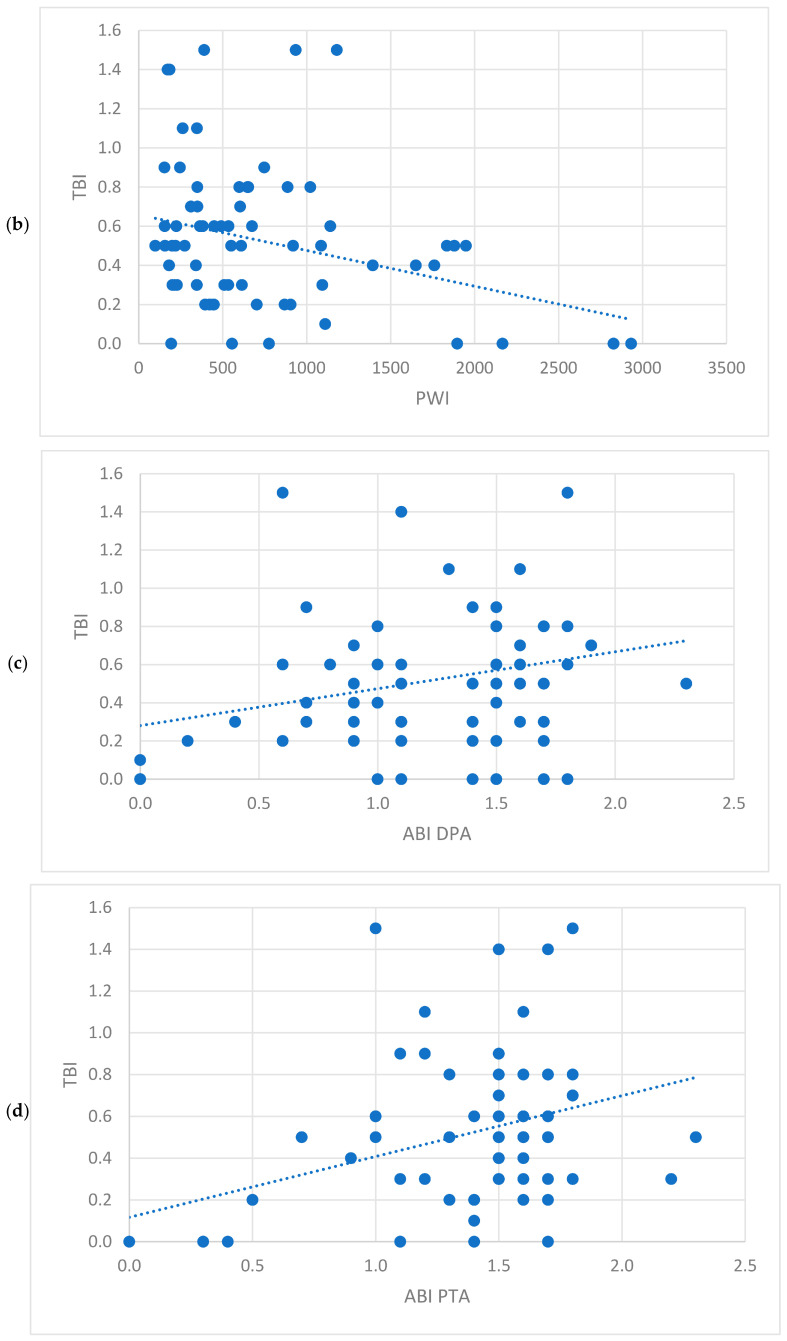

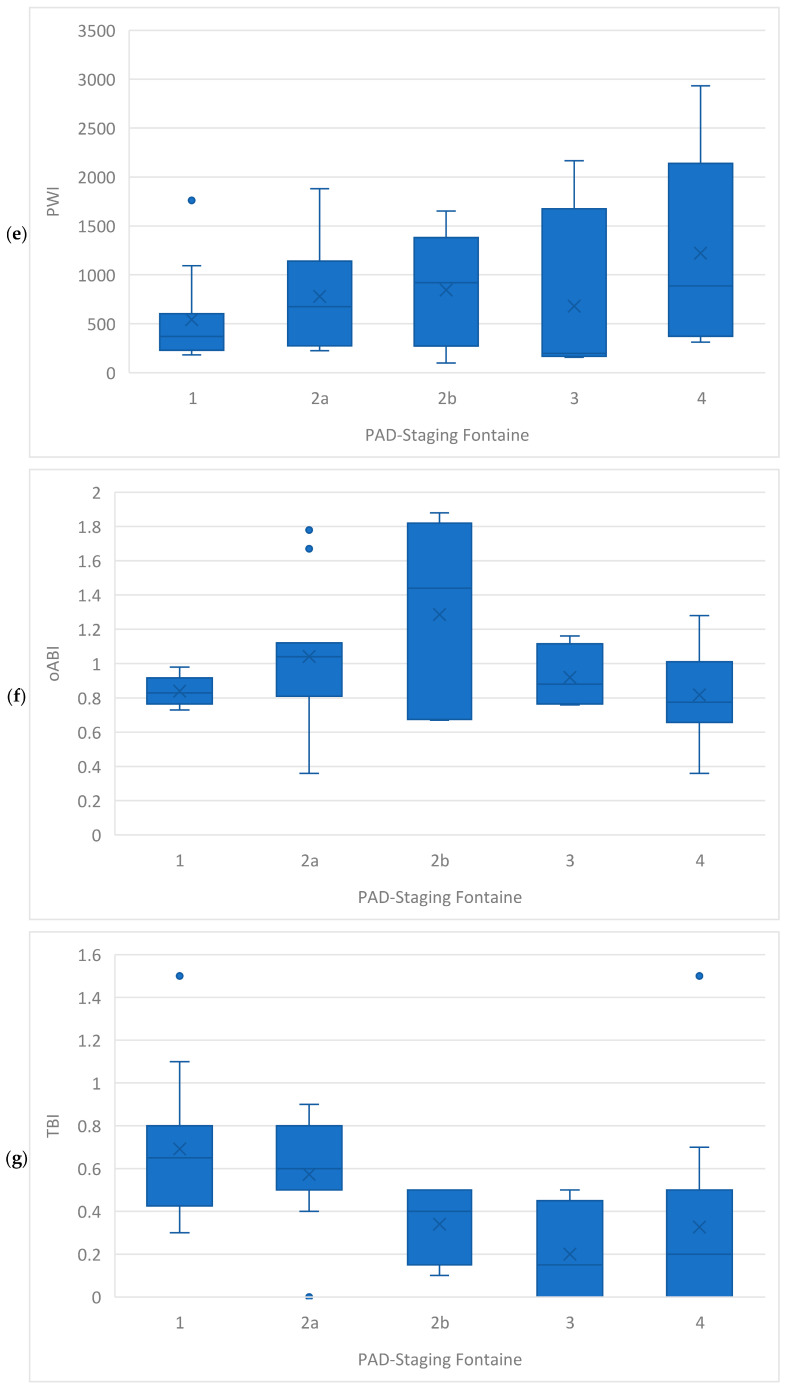
Scatter plot showing the correlation of the study population with media arterial calcification (**a**) between the Toe-Brachial-Index (TBI) and the oscillometric Ankle-Brachial-Index (oABI); (**b**) between the TBI and the Pulse-Wave-Velocity Index; (**c**) between the TBI and the Ankle-Brachial-Index (ABI) of the dorsalis pedis artery (DPA); (**d**) between the TBI and the ABI of the posterior tibialis artery (PTA) and Box-Plott-Diagram showing the correlation of the study population with media arterial calcification (**e**) between the PWI and the peripheral arterial disease staging according to Fontaine (PAD-Staging Fontaine); (**f**) between the oABI and the PAD-Staging Fontaine; (**g**) between the TBI and the PAD-Staging Fontaine.

Analysing all functional tests of the whole population, a correlation with the TBI was seen for all measurements, but the oABI had the weakest correlation (correlation coefficient = 0.236, Figure 2a–d) and the PWI showed the strongest inverse correlation (correlation coefficient = −0.412), which was still only moderate [13]. A strong correlation (correlation coefficient = 0.7) was seen between the ABI of the DPA and the PTA (Table 3). Among the patients without MAC, the strongest correlations were seen between the functional tests (Figure 3 a–d). The TBI again had the strongest correlation with the PWI (correlation coefficient = −0.505), which was also moderate. The oABI again had just weak correlations with all other tests. The PWI had strong inverse correlations with the ABI measured at the DPA (correlation coefficient = 0.631) and the PTA (correlation coefficient = −0.74) (Table 4). Overall, the weakest correlations were seen in the patients with MAC (Figure 4a–g). In these patients, the correlation of the TBI was weak to all other tests, but the strongest for the ABI measured at the PTA (correlation coefficient = 0.338, Figure 4d). The oABI showed no correlation to any testing in this subgroup. But the PWI was weakly inversely correlated to the TBI (Figure 4b, Table 5).

Within the whole population, including both intermittent claudicatio (IC) and chronic limb-threatening ischemia (CLTI) patients, the TBI demonstrated significantly lower values at rest for CTLI patients (0.60 vs. 0.3; *p* < 0.001, Table 5) and significantly higher PWI values (424 vs. 588; *p* = 0.011). No significant differences were shown for oABI (*p* = 0.219). Among patients without MAC, the TBI remained significantly lower in CLTI patients (0.60 vs. 0.40; *p* = 0.005), and PWI was significantly higher (389 vs. 553; *p* = 0.022). There was still no significant difference in measuring the oABI; this was also seen in patients with MAC. Also, the PWI found no significant differences between IC and CTLI patients having MAC (265 vs. 326; *p* = 0.310). But in patients with MAC, the TBI still showed significantly lower values in the CLTI patient (0.3 vs. 0.55; *p* = 0.020).

Within the whole population, correlating the PAD symptom stage according to Fontaine and the functional testing, a weak correlation was seen for the TBI (correlation coefficient = −0.387), PWI (correlation coefficient = 0.327) and ABI of the PTA (correlation coefficient = −0.202), while the oABI and ABI of the DPA did not significantly correlate to the symptom severity (Table 3). Within the group of patients without MAC, a correlation to the PAD symptom stage according to Fontaine was seen for all functional tests but the oABI. The strongest correlation, which was moderate, was seen for the ABI measured at the PTA (correlation coefficient = −0.404), followed by the PWI, which showed a weak correlation (correlation coefficient = 0.386, Figure 3e–g) (Table 4). Among patients with MAC, the TBI correlated moderately with the PAD symptom stage according to Fontaine (correlation coefficient = −0.518, Figure 4g). All other tests did not significantly correlate, but the PWI was close to significance (correlation coefficient = 0.272, *p* = 0.061, Figure 4e) (Table 5).

## 4. Discussion

The prevalence of patients with PAD is continuously increasing, which emphasises the need to evaluate established vascular diagnostics with modern technological possibilities and the development of new procedures [3,14,15].

Especially, patients with DM constitute a high-risk group of evolving PAD, as the prevalence of PAD is twice as high in diabetics as in the rest of the population. In addition, they have an increased risk of rapid disease progression and a more severe etiopathology. Including an increased risk for amputations and an increased mortality risk [16,17,18]. According to current meta-analysis, the risk for major amputations and cardiovascular mortality is 1.5 to 2 times higher than in non-diabetics [16,19,20]. This urges the need for a simple, affordable and reliable diagnostic tool to detect and rule out PAD in DM patients. Our finding that the ABI loses its correlation to the symptom severity of the patients aligns with previous studies showing the ABI is interfered with by MACs [8,21,22]. Furthermore, the finding that MAC weakened correlations between all types of measurements highlights the diagnostic challenges in this subgroup. Although outpatient examinations with ABI and TBI are considered to be effective and time-efficient, human error during the examination must not be disregarded [4,15,23]. Technological improvements in functional diagnostics make the realisation of a fast and reliable solution in outpatient vascular medicine increasingly likely. The TOPP method, which enables the simultaneous measurement of several parameters, might be a possibility to improve the diagnostics workflow [8,21,24,25,26,27,28].

In the study by the research group Horstick et al. 2020, a so-called ‘one-stop-shop’ solution using the automated measurement called TOPP was evaluated in patients with PAD and promising results were obtained regarding the examination procedures using oABI and PWI in a healthier population [1]. The examination of the TOPP method performed in this study was carried out automatically in a time window of just three minutes after laying the patient down on the exam table.

An important aspect in the diagnosis of PAD is the correlation between the functional method used and the severity of the patient’s symptoms. However, this is particularly difficult in diabetic patients, who, compared to the normal population, not only have a lower symptom burden due to more pronounced polyneuropathy, but also due to the limitation of the methods used by MAC [10,18,19,20,29].

Within our study, there were also some differences in our patient population. The significant age difference between the groups (77.2 years vs. 69 years, *p* = 0.001) suggests that MAC is an age-related process or that older patients were exposed to a longer cumulative risk of developing MAC [17,19]. This age distribution could also explain the different symptom presentation, as patients with MAC more frequently presented with more severe stages of the disease. For example, 42.1% of MAC patients reached Fontaine stage IV (ulcers), compared with only the largest proportion of non-MAC patients in stage IIb (37.7%, *p* = 0.033).

Furthermore, patients diagnosed with MAC were found to be more likely to receive vitamin K antagonist therapy. Both older age and vitamin K antagonist use may have contributed to increased arterial stiffness and vascular calcification and may therefore have influenced oABI and PWI measurements independently of PAD severity. In particular, age-related arterial stiffening may affect oscillometric pulse detection, while vitamin K antagonist therapy has been associated with vascular calcification processes [30]. Therefore, the observed differences between patients with and without MAC cannot be attributed exclusively to MAC itself.

In Horstick et al. 2020 [1], this was also difficult and could only be demonstrated in Rutherford groups 2–3 in particular. However, our results show that only the TBI showed a significant correlation to the clinical symptoms of patients with MAC, while the PWI was almost significant as well. In a larger population, a significant correlation might be possible. In contrast, all functional tests besides the oABI show a significant correlation in patients without MAC. These results determine the importance of MAC as a factor influencing measurement accuracy and suggest that, despite their limitations, TBI and PWI might serve as correlates to the clinical symptoms.

The oABI showed in all of the conducted tests the weakest or no correlation to the other functional tests and to the symptom severity of the patients. The divergent performance of oABI and PWI in patients with MAC can be elucidated by the underlying measurement principles. The oABI is based on oscillometric cuff pressure measurement and therefore depends on the compressibility of the ankle arteries. In the presence of MAC, arterial stiffening and incompressibility have been demonstrated to impair oscillometric pressure detection and may lead to falsely elevated or unreliable ABI values [8,15]. This may explain why oABI showed no meaningful correlation with TBI or clinical symptom severity in the MAC subgroup. By contrast, PWI is derived from photoplethysmographic pulse-wave assessment and may reflect distal pulse-wave characteristics rather than cuff-based arterial compressibility alone. Therefore, PWI may be less affected by incompressible ankle arteries, although the correlations observed in this study remained weak and should be interpreted cautiously. This aligns with prior studies, which question the diagnostic value of the oscillometric measured ABI [8,15,22,24,25,26,27,28,31,32,33,34,35,36,37]. Previous studies described that the oABI has a tendency to overestimate ABI values [24,25,26]. This leads to reduced sensitivity for PAD diagnosis, with even the pooled sensitivity in meta-analyses being only 65–69% [27,28]. Furthermore, it should be noted that a marked limitation in the comparability of ABI with oABI can be observed in high-risk populations, such as diabetics with PAD. It can be assumed that arterial calcification and the resulting increased stiffness of the vessels impair both methods, but the stronger the oABI [5,8,21].

For further classifications, we separated the groups of patients with CLTI and IC. To our knowledge, there are no publications investigating the comparison of functional diagnostics in patients with CLTI and IC with and without MAC. In both the overall and the subgroups with and without MAC, the TBI showed consistently significantly lower parameters in CLTI than in IC patients. According to PWI, it shows higher values in the overall group and in CLTI patients without MAC. Furthermore, in patients with MAC, these differences disappear. Here as well, the oABI shows no significant differences between IC and CLTI patients in all measured conditions. Therefore, even in the presence of MAC, the TBI has been shown to be the most sensitive parameter for distinguishing between IC and CLTI.

Although the TBI is considered the gold standard for measuring PAD in patients with MAC [3], it also has significant limitations. The meta-analysis conducted by Tehan et al. 2016 [38] showed that sensitivity values vary greatly, ranging from 45% to 100%, and specificity from 16% to 100%.

The present study has highlighted the diagnostic limitations of oABI in diabetic patients with MAC. While oABI may be considered useful as part of an automated vascular assessment in selected patients, its poor correlation with TBI and symptom severity in the MAC subgroup suggests that it should not be used as a stand-alone diagnostic parameter in this population. In contrast, PWI appeared to be less affected by MAC than oABI and demonstrated a closer relationship with TBI and clinical severity. Despite the correlations being only weak to moderate, these findings indicate that PWI may offer supplementary information on peripheral perfusion, particularly when interpreted in conjunction with TBI.

In patients with MAC, TBI remained the parameter most closely associated with clinical symptom severity, and showed significant differences between IC and CLTI patients. Consequently, the present findings do not support the replacement of TBI with PWI. Instead, PWI should be regarded as a potentially complementary parameter. The TOPP protocol has been demonstrated to exhibit certain advantages in terms of automation and standardisation. These advantages may include a reduction in the dependency on examiners and a concomitant reduction in examination time. However, given the relatively weak correlation between PWI and TBI in the MAC subgroup, the additional diagnostic value of PWI requires confirmation in larger studies. However, using the TOPP method and TBI as a comprehensive screening method enables the expeditious diagnosis and treatment of patients with unknown PAD, thereby mitigating the progression of the disease even in diabetic patients with MAC. But it should be taken into account that a lot of PAD patients with DM have undergone a big toe amputation [39]. This makes it impossible to measure the TBI and TOPP method, therefor should new developments try to measure the distal perfusion on the forefoot and not on a single toe.

## 5. Limitations

The present study is subject to several limitations. The sample size was limited, particularly in the subgroup of patients with MAC, which may have reduced the statistical power to detect weak or moderate associations. As no formal a priori power calculation was performed, the present findings should be interpreted as exploratory. Consequently, non-significant subgroup results should not be interpreted as evidence of the absence of an association.

In addition, the study was monocentric and lacked a blinded element. It is therefore possible that site-specific routines, staff training and device-specific calibration may have introduced systematic bias and limited the generalisability of the results. Furthermore, patients with and without MAC exhibited significant disparities in age and vitamin K antagonist utilisation. The influence of both factors on arterial stiffness and vascular calcification is a potential explanation for the observed effects on oABI and PWI measurements, which appear to be independent of the severity of PAD. Due to the limited sample size, robust multivariable adjustment for these potential confounders was not possible.

Another limitation concerns the definition of MAC, which was determined on the basis of functional ABI values greater than 1.40; no additional imaging, such as plain radiography or ultrasound evidence of incompressible arteries, was used to systematically confirm the diagnosis. Consequently, the possibility of misclassifying patients with incompressible vessels but ABI values within the normal range cannot be completely ruled out. It is recommended that future studies incorporate standardised imaging-based confirmation of MAC.

Finally, the issue of skin pigmentation was not assessed as a statistical variable. As no patients with dark skin pigmentation were included in this cohort, the potential influence of darker skin pigmentation on photoplethysmographic pulse-wave detection and PWI measurements could not be evaluated. Consequently, future studies investigating optical perfusion-based methods should include a more diverse population and document skin pigmentation or sensor signal quality in a standardised manner.

In conclusion, the present study was cross-sectional in nature and did not encompass the assessment of clinically relevant outcomes. These outcomes include, but are not limited to, wound healing, revascularisation, amputation risk, and disease progression. In order to validate the diagnostic value of TOPP-derived parameters, larger, adequately powered multicentre studies are required. Such studies should include standardised imaging-based confirmation of MAC, adjustment for relevant confounders such as age, kidney function, anticoagulant therapy and diabetes-related factors and systematic evaluation of the technical aspects of optical pulse-wave detection, including sensor signal quality and measurement reproducibility. Longitudinal studies are also required to ascertain whether PWI or other TOPP-derived parameters offer supplementary prognostic value in diabetic patients with PAD and MAC.

## 6. Conclusions

In patients without MAC, the TOPP-derived parameters of oABI and PWI exhibited a correlation with ABI and TBI. In patients with MAC, however, TBI remained the most reliable and guideline-recommended functional parameter in the present study. Despite the demonstrated correlation between PWI and TBI, the observed correlation was weak, thus failing to provide robust support for the replacement of TBI with PWI. PWI may, in fact, furnish supplementary information within the context of an automated diagnostic protocol. Conversely, oABI did not demonstrate a correlation with clinical symptom severity in patients with DM, independently of the presence of MAC. Consequently, oABI appears to be an unsuitable parameter in isolation for this patient population. In order to ascertain whether the combination of TBI and TOPP-derived parameters can improve the assessment of PAD severity in diabetic patients with MAC, larger multicentre studies are required.

## Figures and Tables

**Table 1:** Patient characteristics included in the study.

	Pat. with Media Arterial Calcification (n = 38)	Pat. Without Media Arterial Calcification (n = 69)	*p*-Value
**Female gender**	6 (15.8)	19 (27.5)	0.233
**Diabetes mellitus**	38 (100)	69 (100)	1
**Age (years) (SD)**	77.2 (9.9)	69 (14.4)	0.001
**Body surface area**	2.00 (0.22)	1.97 (0.26)	0.789
**Coronary artery disease**	18 (47.4)	33 (47.8)	1
**CKD**	8 (21.1)	13 (18.8)	0.803
**Art. Hypertension**	35 (92.1)	61 (88.4%)	0.743
**Dyslipidemia**	11 (57.9)	30 (55.6)	1
**Previous CVA**	4 (10.5)	2 (2.9)	
**Stroke**	3 (7.9)	2 (2.9)	
**TIA**	1 (2.6)	0	0.194
**Atrial fibrillation**	9 (23.7)	9 (13)	0.183
**Smoking Status**			
**Current**	5 (13.2)	27 (39.1)	
**Former smoker**	13 (34.2)	21 (30.4)	0.012
**HFrEF**			
**Mild**	2 (5.3)	0	
**Moderate**	11 (13.2)	6 (8.7)	
**Severe**	6 (10.5)	2 (2.9)	0.053
**HFpEF**	2 (5.3)	1 (1.4)	0.287
**AAA**	1 (2.6)	8 (11.6)	0.154
**TAA**	1 (2.6)	3 (4.3)	1
**PAD Stage according to** **Fontaine**			
**I**	7 (18.4)	10 (14.5)	
**IIa**	7 (18.4)	13 (18.8)	
**IIb**	4 (10.5)	26 (37.7)	
**III**	4 (10.5)	5 (7.2)	
**IV**	16 (42.1)	15 (21.7)	0.033

Dates are presented as the number characteristics as reported. Values in parentheses are percentages unless indicated otherwise. CKD = chronic kidney disease; CVA = cerebrovascular accident; TIA = transient ischemic attack; HFrEF = heart failure with reduced ejection fraction; HFpEF = heart failure with preserved ejection fraction; AAA = abdominal aortic aneurysm; TAA = thoracic aortic aneurysm; PAD = peripheral arterial disease.

**Table 2:** Used medication of the study population.

	Pat. with Media Arterial Calcification (n = 38)	Pat. Without Media Arterial Calcification (n = 69)	*p*-Value
**Statin**	31 (81.6)	60 (87)	0.572
**Ezetimib**	10 (26.3)	17 (24.6)	1
**ASA**	20 (52.6)	48 (69.6	0.096
**Clopidogrel**	6 (15.8)	17 (24.6)	0.334
**Ticagrelor**	1 (2.6)	4 (5.8)	0.654
**Prasugrel**	0	2 (2.9)	0.538
**DOAC**	15 (39.5)	18 (26.1)	0.190
**Vitamin K antagonist**	9 (23.7)	3 (4.3)	**0.** **004**

Dates are presented as the number of medications as reported. Values in parentheses are percentages unless indicated otherwise. ASA = acetylsalicylic acid, DOAC = direct oral anticoagulant.

**Table 3:** Correlations between the TBI, ABI, oABI, and PWI functional tests among themselves and with the clinical PAD stage within the entire study population.

Correlations								
			TBI	ABI DPA	ABI PTA	oABI	PWI	PAD Stage
Spearman’s rho	TBI	Correlation Coefficient	1.000	0.259 **	0.273 **	0.236 **	−0.412 **	−0.387
		Sig. (2-tailed)	-	<0.001	<0.001	<0.001	<0.001	<0.001
		N	210	208	210	209	209	129
	ABI DPA	Correlation Coefficient	0.259 **	1.000	0.700 **	0.140 *	−0.315 **	−0.073
		Sig. (2-tailed)	<0.001	-	<0.001	0.044	<0.001	0.415
		N	208	210	210	209	209	126
	ABI PTA	Correlation Coefficient	0.273 **	0.700 **	1.000	0.196 **	−0.338 **	−0.202
		Sig. (2-tailed)	<0.001	<0.001	-	0.004	<0.001	0.022
		N	210	210	212	211	211	128
	oABI	Correlation Coefficient	0.236 **	0.140 *	0.196 **	1.000	−0.271 **	−0.143
		Sig. (2-tailed)	<0.001	0.044	0.004	-	<0.001	0.110
		N	209	209	211	211	211	127
	PWI	Correlation Coefficient	−0.412 **	−0.315 **	−0.338 **	−0.271 **	1.000	0.327
		Sig. (2-tailed)	<0.001	<0.001	<0.001	<0.001	-	<0.001
		N	209	209	211	211	211	127

** Correlation is significant at the 0.01 level (2-tailed). * Correlation is significant at the 0.05 level (2-tailed).

**Table 4:** Correlations between the TBI, ABI, oABI, and PWI functional tests among themselves and with the clinical PAD stage within the population without media arterial calcification.

Correlations ^a^								
			TBI	ABI ADP	ABI ATP	oABI	PWI	PAD Stage
Spearman’s rho	TBI	Correlation Coefficient	1.000	0.437 **	0.446 **	0.310 **	−0.505 **	−0.264
		Sig. (2-tailed)	-	<0.001	<0.001	<0.001	<0.001	<0.001
		N	138	136	138	137	137	79
	ABI ADP	Correlation Coefficient	0.437 **	1.000	0.639 **	0.334 **	−0.631 **	−0.299
		Sig. (2-tailed)	<0.001	-	<0.001	<0.001	<0.001	0.008
		N	136	137	137	136	136	78
	ABI ATP	Correlation Coefficient	0.446 **	0.639 **	1.000	0.288 **	−0.740 **	−0.404
		Sig. (2-tailed)	<0.001	<0.001	-	<0.001	<0.001	<0.001
		N	138	137	139	138	138	80
	oABI	Correlation Coefficient	0.310 **	0.334 **	0.288 **	1.000	−0.348 **	−0.151
		Sig. (2-tailed)	<0.001	<0.001	<0.001	-	<0.001	0.185
		N	137	136	138	138	138	79
	PWI	Correlation Coefficient	−0.505 **	−0.631 **	−0.740 **	−0.348 **	1.000	0.386
		Sig. (2-tailed)	<0.001	<0.001	<0.001	<0.001	-	<0.001
		N	137	136	138	138	138	79

** Correlation is significant at the 0.01 level (2-tailed). ^a^ MS = 0.

**Table 5:** Comparison of PWI, oABI and TBI between patients with intermittent claudication and chronic limb-threatening ischemia. Analyses were performed for the overall study population and subpopulations of patients with and without MAC. Values are presented as median with interquartile range.

		IC	CTLI	*p*-Value
PWI	Overall	424 [249–746]	588 [330–1180]	0.011
	No MAC	389 [248–739]	553 [328–1195]	0.022
	MAC	265 [512–852]	326 [615–1133]	0.310
oABI	Overall	0.88 [0.79–1.00]	0.83 [0.73–0.98]	0.219
	No MAC	0.87 [0.79–1.00]	0.84 [0.75–0.93]	0.356
	MAC	0.90 [0.77–1.03]	0.83 [0.73–0.99]	0.334
TBI	Overall	0.60 [0.40–0.70]	0.30 [0.20–0.70]	<0.001
	No MAC	0.60 [0.40–0.70]	0.40 [0.28–0.70]	0.005
	MAC	0.55 [0.40–0.78]	0.30 [0.20–0.68]	0.020

## Data Availability

The data supporting the findings of this study are available upon reasonable request from the Department for Angiology, Centre of Cardiology, University Medical Centre Mainz. Due to institutional and data protection regulations, the data are not publicly available.
